# Under the Microscope: Single-Domain Antibodies for Live-Cell Imaging and Super-Resolution Microscopy

**DOI:** 10.3389/fimmu.2017.01030

**Published:** 2017-08-24

**Authors:** Bjoern Traenkle, Ulrich Rothbauer

**Affiliations:** ^1^Pharmaceutical Biotechnology, Eberhard Karls University Tuebingen, Tuebingen, Germany; ^2^Natural and Medical Sciences Institute at the University of Tuebingen, Reutlingen, Germany

**Keywords:** nanobodies, intrabodies, chromobodies, cytoskeleton, live-cell imaging, high-content imaging, super-resolution microscopy

## Abstract

Single-domain antibodies (sdAbs) have substantially expanded the possibilities of advanced cellular imaging such as live-cell or super-resolution microscopy to visualize cellular antigens and their dynamics. In addition to their unique properties including small size, high stability, and solubility in many environments, sdAbs can be efficiently functionalized according to the needs of the respective imaging approach. Genetically encoded intrabodies fused to fluorescent proteins (chromobodies) have become versatile tools to study dynamics of endogenous proteins in living cells. Additionally, sdAbs conjugated to organic dyes were shown to label cellular structures with high density and minimal fluorophore displacement making them highly attractive probes for super-resolution microscopy. Here, we review recent advances of the chromobody technology to visualize localization and dynamics of cellular targets and the application of chromobody-based cell models for compound screening. Acknowledging the emerging importance of super-resolution microscopy in cell biology, we further discuss advantages and challenges of sdAbs for this technology.

Reflecting the importance of cellular imaging, microscopic technologies ranging from wide-field to super-resolution microscopy are applied in nearly every cell-biological laboratory. Along with recent developments such as high-content live-cell imaging or super-resolution microscopy, there is a concomitant need for advanced labeling strategies to visualize cellular components in physiologically meaningful states. Here, we review recent progress in the development of camelid-derived single-domain antibodies (sdAbs) for live-cell imaging and super-resolution microscopy.

## sdAbs for Live-Cell Imaging

**Figure 1:**
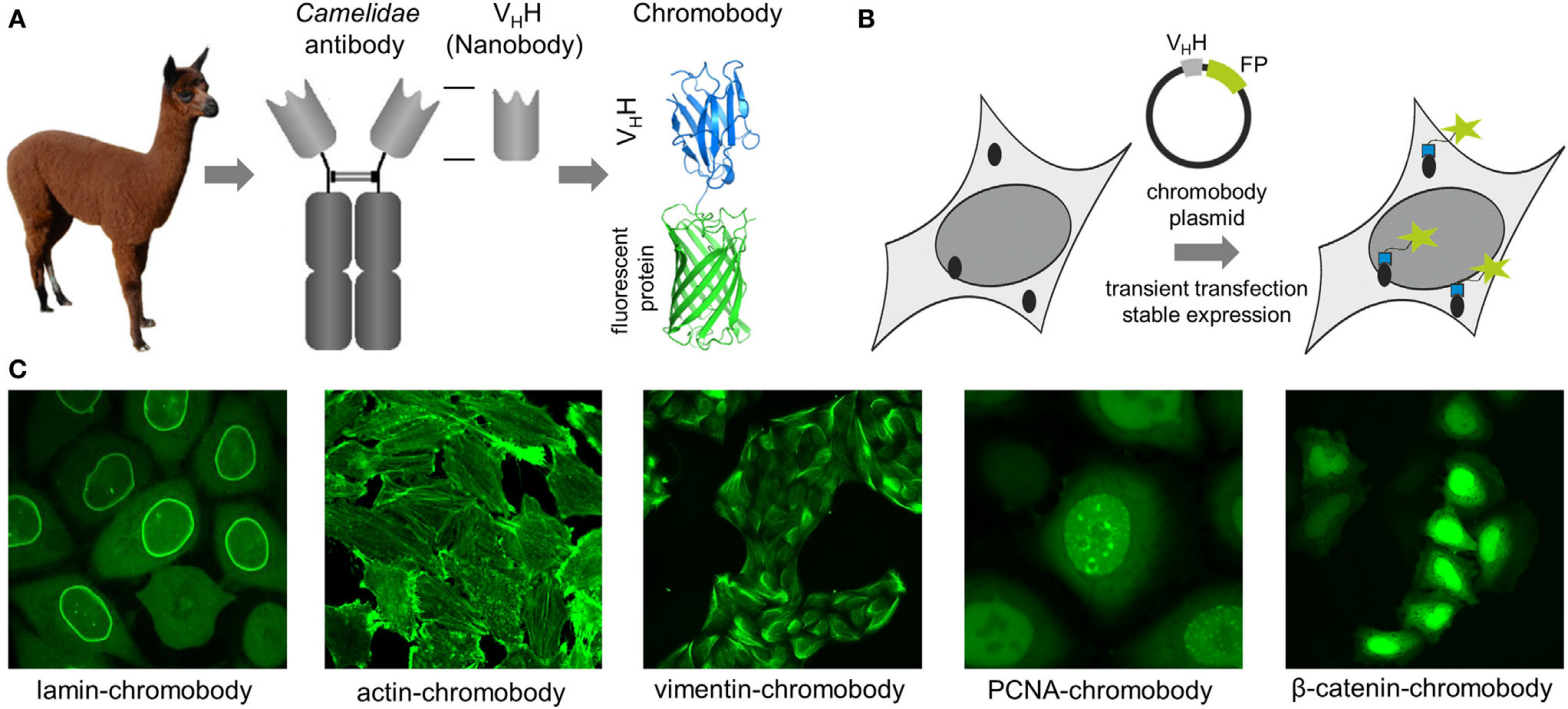
**(A)** Schematic representation of a chromobody derived from a single-domain antibody of *Camelidae*. **(B)** Illustration of intracellular antigen binding of chromobodies followed by introduction and expression of DNA-encoded chromobody expression constructs. **(C)** Representative images of endogenous cellular structures visualized by recently developed chromobodies directed against lamin A, ACTB, vimentin, proliferating cellular antigen (PCNA), and β-catenin in living cells.

Antigen staining with conventional antibodies is still the most popular approach to image native cellular antigens, but due to chemical fixation of the cells it is not suitable to monitor dynamic processes. For visualization in living cells, proteins can be fused either to self-labeling enzymes (SNAP-, Halo-, or CLIP-tag) or fluorescent proteins (FP) (1–5). However, addition of such large protein tags (~20–25 kDa) to the N- or the C-terminus may affect the expression level, activity, and localization, and for some targets, it was shown that expression of the corresponding fusion protein affects cellular morphology or function (6–8). To avoid genetic modification, intracellularly functional binding molecules (intrabodies) have been developed to visualize endogenous targets. While some intrabodies are based on non-antibody scaffolds like peptides, monobodies, or designed ankyrin repeat proteins (9–12), most intrabodies are derived from immunoglobulins (IgGs) comprising a variable heavy (VH) and variable light domain, artificially linked to form a single-chain variable fragment (scFv) (13–15). Due to their compact structure, small size, high stability, and solubility, sdAb fragments (V_H_Hs, nanobodies) from camelids (16) provide beneficial properties for intracellular applications (11, 17). However, only nanobodies which retain a binding-compatible conformation in the absence of the conserved disulfide bond connecting frameworks 1 and 3 are functionally expressed in live cells, as disulfide bridges are not formed in the reducing environment of the cytoplasm. Such binders have to be selected experimentally, whereas nanobodies comprising additional disulfide bonds, e.g., to stabilize complementarity-determining regions forming the paratope can be excluded *a priori* based on their DNA sequence. Nowadays, numerous protocols and synthetic libraries are available which facilitate the selection of intracellular nanobodies (18–24). For visualization of endogenous antigens, nanobodies were genetically fused to fluorescent proteins and introduced as DNA-encoded expression constructs in living cells. Reflecting their chimeric structure these constructs were termed “chromobodies” (25) (Figures 1A,B).

In an initial study, a red fluorescent chromobody directed against GFP was generated. Fluorescence co-localization analysis of living cells expressing the GFP-chromobody in combination with different GFP-labeled marker proteins (components of the cytoskeleton, nuclear lamina, or chromatin) revealed a high overlap of the fluorescence intensities of antigen and chromobody. Besides functional expression in the cytoplasm, the GFP chromobody was shown to enter the nucleus, where it traces dynamic changes of cellular antigens (e.g., H2B-GFP) throughout different stages of the cell cycle (25). Since its first description, the GFP-chromobody has been widely used for multiple functional and imaging applications ranging from targeted relocalization (26–28), induced proteasomal degradation (29, 30), to high-throughput translocation assays (31) of GFP-tagged proteins. While the GFP-chromobody became a unique tool to study GFP-tagged proteins in many facets, numerous chromobodies directed against native proteins have been generated during the last decade.

## Chromobodies to Visualize the Cytoskeleton

Chromobodies that visualize, but do not disturb the cytoskeleton network, are highly desirable for live-cell imaging as many of the cytoskeletal proteins become only partially integrated into native structures when administered as FP fusions (7, 32–34). To date, numerous chromobodies targeting proteins involved in the formation of the nuclear lamina, actin, and intermediate filaments have been described. A lamin-chromobody was identified and stably introduced in human cell lines (Figure 1C) (35). Fluorescent recovery after photobleaching (FRAP) analysis showed that the lamin-chromobody binds very transiently, which does not interfere with the functional redistribution of the nuclear lamina (25). Live-cell imaging of the chromobody signal revealed the typical nuclear rim structure and monitors its disintegration during mitosis or upon compound-mediated induction of apoptosis (36). For *in vivo* labeling of the actin cytoskeleton, an actin-chromobody with a similar highly transient binding mode was generated (Figure 1C) (37). Originally selected against mammalian ACTB, it also recognizes F-actin in parasites, zebrafish, or plant cells (37–40). Not disturbing actin dynamics by steric hindrances or stabilizing effects, the actin-chromobody provides distinct benefits over other labeling approaches such as lifeact-GFP (10) or SiR-Act (41). Thus, the actin-chromobody was used to track the movement of Golgi bodies along actin filaments in tobacco leaf cells. Compared to lifeact-GFP, a more complex movement pattern of the organelles was detectable, indicating that the transiently binding chromobody has only a minor effect on actin dynamics in those plant cells (40). In a recently published report, Periz et al., used the actin-chromobody to visualize actin for the first time in *Toxoplasma gondii*. Previous attempts with other labeling approaches have failed due to the fast turnover of F-actin in those parasites. Since the actin-chromobody preferentially labels filamentous actin the dynamics of the extensive actin network that connects parasites within the parasitophorous vacuole becomes visible and the exchange of vesicles between individual parasites could be monitored (38). In another approach the actin-chromobody was directed to the nucleus of mammalian cells. Following the chromobody signal in stably expressing NIH3T3 cells a fast formation of actin fibers within the nucleus in response to cellular treatment with soluble fibronectin was observed by time-lapse imaging (42). Moreover, the generation of an actin-chromobody expressing zebrafish established the first application of chromobodies in a vertebrate. Embryos ubiquitously expressed the actin-chromobody were raised to adulthood demonstrating that this intrabody does not interfere with normal animal development. Live imaging of whole zebrafish at various developmental stages revealed distribution and dynamics of actin in different cell types including embryonic muscle fibers, migrating primordial cells, epidermal cells, macrophages, or xanthophores and provides novel insights into processes such as wound healing or neuronal development (37).

Addressing another cytoskeletal target, we recently have generated a vimentin-specific chromobody (VB6-chromobody) to label major intermediate filaments *in vivo* (Figure 1C) (43). In addition to its role as an essential component of the cytoskeletal network, vimentin is a biomarker of epithelial–mesenchymal transition (EMT), a highly dynamic process involved in the initiation of metastasis and cancer progression. Thus, a lung cancer cell model stably expressing the VB6-chromobody was established and dynamic changes of endogenous vimentin were monitored. Upon treatment with TGF-β as an inductor of EMT, the chromobody signal revealed the incremental formation of vimentin fibers over time, starting from the nucleus toward the cellular periphery while upon RNAi-mediated vimentin depletion we observed an increasingly diffusible distribution of the chromobody in live cells. Based on these findings, we established a phenotypic readout for high-throughput live-cell imaging and quantified dose- and time-dependent effects of vimentin-modulating compounds as novel potential inhibitors of EMT (43, 44). In summary, to date, numerous cytoskeleton-specific chromobodies are available. They provide a promising approach for labeling these components in living cells and can be implemented in phenotypic screening approaches using 2D- or 3D-chromobody cell models (36, 44) or whole organism (37). However, no intracellularly functional tubulin-chromobody was reported so far, which would ideally complement this set of cytoskeletal probes.

## Chromobodies Visualizing Nuclear Components

Chromobodies directed against nuclear factors have also found their way into live-cell imaging. Visualizing the dynamic appearance of distinct nuclear foci, formed by the native proliferating cell nuclear antigen (PCNA), a PCNA chromobody allows a detailed time-lapse analysis of S phase progression and quantitative live imaging of endogenous DNA replication in human cells (Figure 1C) (45). The potential of the PCNA chromobody to monitor the cell cycle using real-time high-throughput imaging was recently combined with an enzymatic determination of dead cell protease activity in corresponding cell culture supernatants. By this cell cycle modulators derived from a compound library which show low cellular toxicity were identified (46). In a similar setting, a PARP1 chromobody was used to visualize recruitment of endogenous PARP1 to DNA-damaged sites. The possibility to trace the characteristic relocalization of PARP1 from nucleoli to nucleoplasm following the chromobody signal constitutes a novel cell-based screening for rRNA transcription inhibitors or DNA-damaging agents using a translocation-specific, real-time imaging approach (47). Addressing the heterodimer formed by H2A–H2B histones, a chromobody (chromatibody) was developed for chromatin labeling of a wide variety of cell lines ranging from yeast to human. Although this chromobody shows a high affinity, its expression does not affect normal cell cycle progression. Moreover, similar to the actin-chromobody, introduction of the H2A–H2B chromobody in a transgenic *Drosophila* model has no influence on normal development underlining the functionality of chromobodies for non-invasive imaging of native targets in whole organisms (48).

All previously described chromobodies address structurally defined antigens (fibers, spots, etc.). A more challenging approach is to probe soluble cellular components. Recently, a chromobody specific for the Wnt signaling component β-catenin in its hypo-phosphorylated state was developed and stably introduced into HeLa cells. This chromobody cell line was used to monitor cytoplasmic accumulation and nuclear translocation of endogenous β-catenin in response to compound treatment (Figure 1C). This study additionally describes a previously unappreciated dependency of the chromobody level on the amount of its antigen and demonstrates that the chromobody signal can be utilized to trace quantitative changes of cellular β-catenin levels in real time (49).

Finally, a conformation-specific chromobody which visualizes GPCR trafficking from the plasma membrane to endosomes should be highlighted. Starting from a nanobody selectively recognizing the activated β2-adrenoceptor (β2-AR) (50), Irannejad et al. generated a GFP fusion of this binder (Nb80-GFP). Upon activation of β2-AR with isoprenaline, this chromobody was rapidly recruited from a diffuse fraction to the plasma membrane. Continued time-lapse imaging showed a displacement of the chromobody when β-arrestin binds to internalized β2-AR and relocalization of the chromobody signal to β2-AR-containing endocytic vesicles further revealed a restoration of target binding once endosomes became uncoated (51). This study impressively demonstrates the potential of chromobody-based probes to visualize dynamic conformational changes of signaling proteins with high spatiotemporal resolution in living cells.

The aforementioned examples provide a rather short overview of recent advances of the chromobody technology. For the sake of brevity, many other chromobodies and applications thereof, e.g., to visualize viral morphogenesis (52), actin-binding proteins (19) or to manipulate native targets and structures (53, 54) in living cells are only mentioned briefly here.

Like for any other molecular probe applied for live-cell imaging, influence of chromobody expression has to be carefully evaluated. Especially, impact on antigen mobility or displacement of natural interaction partners has to be considered. This can be addressed, e.g., by selecting transiently binding chromobodies, detectable by FRAP analysis (23, 25, 37, 43), or chromobodies addressing inert epitopes, which can further be analyzed by intracellular immune-precipitations to monitor interactors co-precipitating with the antigen (49). Stable chromobody cell lines further requires detailed evaluation of, e.g. morphology, proliferation, and signaling pathways the target is involved in (43). To date, most chromobodies visualizes proteins present in considerable amounts in the cell. Introduced as genetically-encoded constructs their expression is only partially adjustable and signal of bound chromobodies is affected by the diffuse signal derived from non-bound ones. Gross et al. recently described an elegant approach to adjust the level of an intrabody by fusing it to a DNA-binding KRAB domain which induces a dynamic feedback mechanism transcriptionally repressing the generation of non-target-bound intrabody (9). Another option is to generate destabilized chromobodies. By adding a destruction motif such as PEST domains (55) or introducing distinct point mutations in the framework regions (56), the cellular turnover of chromobodies can be increased. Since we and others have observed that chromobodies are stabilized upon antigen binding (49, 56), such modifications might be suitable to improve the detection of low abundant components within living cells.

In summary, chromobodies are versatile probes to monitor expression and dynamics of endogenous proteins *in vivo*. Their ability to visualize antigens without affecting their function makes them ideally suited for real-time imaging of cellular processes and redistribution assays. To combine *in cellulo* imaging with functional studies, chromobodies which interfere with distinct protein functions or interactions can be selected. Such intrabodies would offer new perspectives for target identification, validation, and visualization in living cells.

## sdAbs for Super-Resolution Imaging

With structured illumination microscopy (SIM) (57), stimulated emission depletion microscopy (STED) (58) and single-molecule localization techniques such as photoactivation localization microscopy (59) or stochastic optical reconstruction microscopy (STORM) (60), highly advanced methods are now available to image biological samples at resolutions below the diffraction limit of light (61, 62). In parallel, novel labeling strategies and improved affinity probes for SRM are developed (63–65). However, to date SRM-compatible fluorophores are most commonly delivered by expression of (photoactivatable) FPs or indirect labeling using secondary antibodies conjugated to organic dyes. Antibodies as relatively large molecules (150 kDa, 10–15 nm) can interfere with the achievable resolution as they displace the fluorophore from the target and introduce a so-called “linkage error” (66). Obviously, the 10 times smaller nanobodies (15 kDa, 2–4 nm) are predestined to overcome these issues as they have better access to intracellular antigens and can be easily conjugated to fluorophores either by chemical coupling or enzymatic labeling (67). Despite their clear advantages for the field, nanobodies are still at an early stage as novel labeling probes for super-resolution microscopy.

The first nanobody applied for super-resolution was the GFP nanobody in SIM studies (68). SIM requires very photostable labeling and GFP fusions often suffer from massive photobleaching during extended image acquisition. Upon binding to the high-affinity GFP nanobody (25) coupled to green fluorescent organic dye fluorescent intensities of individual GFP fusions can now be “boosted,” to restore and increase the signal in the green channel. Combining SIM with ATTO488-conjugated GFP nanobodies, Guizetti et al. regain fluorescence of GFP-labeled components of endosomal sorting complex required for transport-III and obtained insights in the organization of the intracellular cortical constriction zone at the nanoscale (68). For single-molecule localization microscopy (SMLM), Ries et al chemically coupled the GFP and RFP nanobody developed by ChromoTek to AlexaFluor 647 (AF647) and AlexaFluor 700. They stated that nanobody-mediated targeting of organic dyes to FP fusions combines molecular specificity of genetic tagging with high photon yield of organic dyes and minimal linkage error. By staining of tubulin-YFP in Ptk2 cells with the GFP nanobody, they achieved a high-density labeling of microtubules with a full width half maximum of ~30 nm for individual filaments, which is in accordance with the reported microtubule diameter of ~25 nm. Additionally, they showed that the small GFP nanobody is able to penetrate the permeabilized cell wall of intact yeast cells without generating spheroblasts. Thus, they were able to perform STORM imaging of multiple endogenous GFP fusions derived from a haploid genomic library of *S. cerevisiae* (69).

Due to its applicability for nanoscopy of widely available GFP fusions, the GFP nanobody becomes a very popular tool for SMLM. Recently, the GFP nanobody was used to explore the structural background of information transmission in the nervous system. For localization microscopy of nanoclusters in the pre- and postsynaptic neurons the endogenous postsynaptic scaffolding protein PSD-95 was replaced by a GFP-tagged knockdown rescue variant of PSD-95 and labeled with the GFP nanobody conjugated at a 1:1 ratio with ATTO647. This provided a detailed insight in distributions of proteins mediating vesicle priming in the presynaptic active zone in relation to postsynaptic membranes marked by PSD-95 (70).

While GFP- or RFP nanobodies are highly suitable for SRM of FP fusions in fixed and also live-cells when coupled to quantum dots (71), this strategy relies on the correct expression of FP fusions and does not cope with problems arising from overexpression, mislocalization or dysfunction (6, 8). Thus, nanobodies directed against smaller and inert peptide tags could be advantageous. With the BC2 nanobody, we recently reported the first peptide-binding nanobody which is suitable for cellular imaging (72). Upon chemical conjugation to organic dyes we visualized BC2-tagged proteins in various cellular compartments including the cytoplasm or the nucleus using wide-field or confocal microscopy (72). Further adaption of the BC2 nanobody to generate a SRM-compatible labeling probe is currently under development in our group.

To avoid any interferences derived from the addition of protein- or peptide tags, nanobodies targeting native proteins would be ideally suited for SRM. Considering that only a very few scientific groups or companies are currently developing nanobodies for SRM, only three examples of target-specific nanobodies can be mentioned here. Pleiner et al. have generated a set of nanobodies addressing various components of the nuclear pore complex (NPCs) of *Xenopus laevis*. For stoichiometric dye conjugation, they exchanged individual amino residues within the framework regions with single cysteines and performed maleimide coupling of AF647. They stated, that in comparison to NHS-mediated labeling, the site-specific conjugation leads to a better signal-to-noise ratio as it decreases unspecific background derived from dysfunctional or hydrolyzed NHS-coupled nanobodies. The AF647-conjugated nanobodies were successfully applied for STORM imaging of nuclear pore components including Nup93, Nup98, and Nup153. A detailed insight in the organization of these nuclear pore forming proteins can be deduced from the obtained images (73). In another example, two nanobodies against endogenous β-tubulin have been generated. STORM imaging with the newly identified anti-tubulin nanobodies in comparison to a primary anti-tubulin antibody either directly labeled or detected by a secondary antibody revealed significant differences in resolving bundled microtubules in various cell lines. While individual microtubules were resolvable with both nanobodies, none of the antibody-mediated labeling approaches led to a successful resolution of bundled microtubules. On quantitative level, the diameter of densely labeled microtubules was determined with ~40 nm for the anti-tubulin nanobodies and ~54 to ~62 nm for the directly conjugated primary anti-tubulin antibody or the primary/secondary antibody approach, respectively. From those findings, the authors concluded that conventional antibody labeling displaces the fluorophore on average ~12.5 nm from the microtubule whereas nanobody labeling reduces this distance to less than 2.5 nm (74).

**Figure 2:**
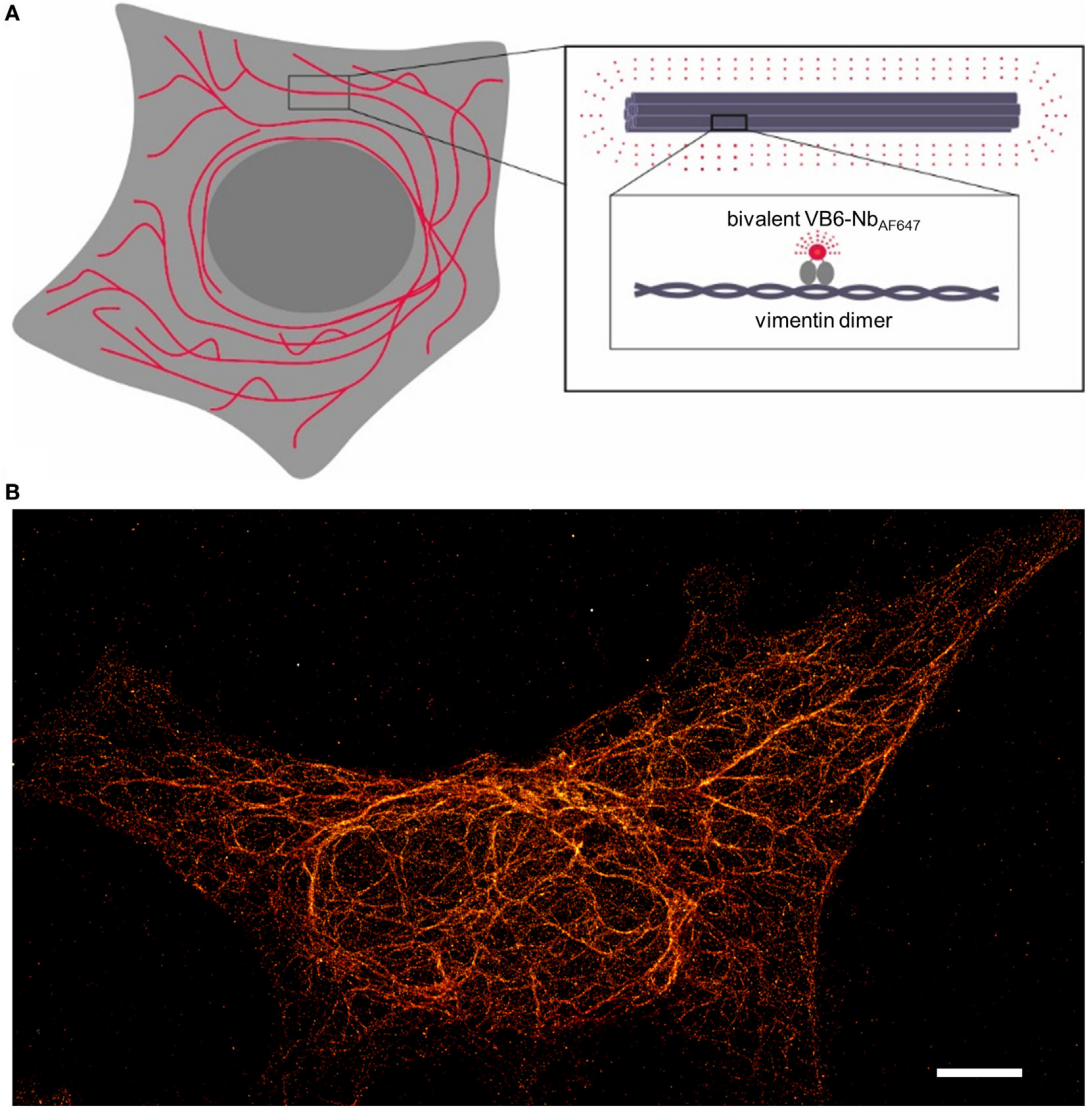
Illustration of the nanobody-based labeling strategy for stochastic optical reconstruction microscopy (STORM) of the native vimentin network. **(A)** Schematical depiction of the bivalent VB6 (bivVB6)-Nb-labeled vimentin network. The boxed regions outline the organization of individual vimentin molecules into larger fibers and highlight the detection of dimeric vimentin with the fluorescently labeled bivalent VB6-Nb (bivVB6-Nb_AF647_). **(B)** Representative STORM image of a HeLa cell, stained with the bivVB6-Nb_AF647_. Scale bar, 5 μm.

Given the fact that our recently developed bivalent VB6 (bivVB6) nanobody also detects native vimentin structures when it is applied as a dye-conjugated labeling probe (43), we further evaluated this bivalent nanobody format for SRM. Thus, we performed site-directed conjugation of VB6-Nb with AF647. STORM images impressively show a high resolution of the vimentin network in mammalian cells indicating that the bivVB6-Nb nanobody is also suitable for SRM (Figure 2, own work).

In summary, nanobodies in combination with site-specific and quantitative fluorescent labeling will be crucial for SRM aiming at detailed structural analysis or determination of absolute protein copy numbers. Although only a limited number of nanobodies are available for SRM, the presented examples excelled in SRM and well-defined cellular structures such as the vimentin network, NPCs, or microtubules labeled with nanobodies have now become benchmarks for many new advancements of SRM. In contrast to conventional poly- or monoclonal antibodies, nanobodies are reliably producible in high yields with a standard quality. Thus, it is conceivable that they will help to avoid current uncertainties regarding antigen labeling and facilitate the reproducibility of results between laboratories and publications. In combination with other approaches developed to deliver bright organic dyes to defined cellular structures such as point accumulation for imaging in nanoscale topography (75), bicyclic peptides (76), or aptamers (77), nanobodies perfectly complement the portfolio of new and reliable labeling probes for super-resolution imaging.

## Author Contributions

UR and BT have jointly written the manuscript and prepared the figures.

## Conflict of Interest Statement

UR is shareholder of ChromoTek GmbH. BT declares that the research was conducted in the absence of any commercial or financial relationships that could be construed as a potential conflict of interest.
